# Analyzing the roles of some species of arthropods in the transmission of the Middle East respiratory syndrome coronavirus

**DOI:** 10.1002/vms3.717

**Published:** 2022-02-01

**Authors:** Maged Gomaa Hemida, Mohammad Al‐Sabi, Mohammed Alhammadi, Faisal Almathen, Abdelmohsen Alnaeem

**Affiliations:** ^1^ Department of Microbiology College of Veterinary Medicine King Faisal University Al‐Ahasa Saudi Arabia; ^2^ Faculty of Veterinary Medicine Department of Virology Kafrelsheikh University Kafrelsheikh Egypt; ^3^ Department of Public Health and Animal Husbandry Veterinary Medicine King Faisal University Hofuf Saudi Arabia; ^4^ Department of Clinical Sciences College of Veterinary Medicine King Faisal University Al‐Ahasa Saudi Arabia

**Keywords:** Culex, Culicoides, dromedary camels, MERS‐CoV, Musca domestica, nasal swabs, real‐time PCR, rectal swabs, RNA, Stomoxys, transmission

## Abstract

**Background:**

The Middle East Respiratory Syndrome coronavirus (MERS‐CoV) is still listed on the WHO Research and Development Blueprint of emerging pathogens. Dromedary camels remain the only known animal reservoir of the virus. The animal‐to‐animal as well as the animal‐to‐human transmission in the MERS‐CoV cycles were reported. However, many aspects of these transmission chains are not well studied. One of these directions is the potential roles of various species of arthropods in the transmission of the virus.

**Objectives:**

The main goal of the current work was to study the roles of several species of arthropods in the transmission of MERS‐CoV.

**Methodology:**

To achieve this goal, we identified some MERS‐CoV naturally infected dromedary camel populations. We conducted a longitudinal study among these animals for more than 2 months. This was done by repeated testing of nasal swabs biweekly from some selected animals in this population for the presence of MERS‐CoV‐RNAs by real‐time PCR. During the duration of this study, we collected several species of arthropods (*Culicoides*, *Stomoxys*, *Musca domestica* and some *Culex* species) that shared the habitat and were circulating in this farm during this longitudinal study.

**Results:**

Our results showing, despite the detection of the viral RNAs in some animals throughout this study, none of the examined species of arthropods tested positive for the viral RNA.

**Conclusions:**

These results are suggesting that at least the tested species of arthropods may not play roles in the transmission of MERS‐CoV. However, more large‐scale studies are required to explore any potential roles of arthropods in the transmission cycle of MERS‐CoV.

## Graphical abstract

• Identification of some index MERS‐CoV naturally infected dromedary camel population.

• A longitudinal follow‐up study for MERS‐CoV naturally infected dromedary camels based on repeated testing of nasal swabs from some animals revealed the contentious circulation of the virus in these animals throughout the duration of the study.

• The absence of any detectable MERS‐CoV‐RNAs in some collected arthropods shared the habitat with the infected camel population strongly suggests the lack of transmission of MERS‐CoV through the tested species of arthropods.

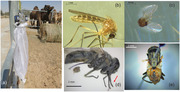

## 1 INTRODUCTION

MERS‐CoV is one of the recently identified coronavirus zoonotic infections, which is an ideal example of the One Health concept. There are 2580 human infections of MERS‐CoV that have been reported to the WHO from 27 countries as of 6 April 2021 (WHO, 22021). Dromedary camels play key roles in the transmission and sustainability of the virus in the environment (Azhar et al., 2014). MERS‐CoV infections in dromedary camels resulted in the development of some clinical diseases and pathological changes in the respiratory organs of some affected animals either under the experimental or under the natural field infection (Adney et al., 2014, Alnaeem et al., 2020). The dynamics and the transmission of MERS‐CoV among dromedary camels were reported in many countries especially in the Arabian Peninsula (Lau et al., 2016, Nowotny & Kolodziejek, 2014, Raj et al., 2014). Although the transmission of the MERS‐CoV between animals and from animals to humans was reported (Azhar et al., 2014, Nowotny & Kolodziejek, 2014, Raj et al., 2014). Some progress was made in the direction of our understanding of the methods of MERS‐CoV transmission between animals and from animals to humans. The viral particles and their RNAs were detected in the camel seminal plasma, saliva, and breath as well as in the circulating air of some naturally infected dromedary farms (Azhar et al., 2014, Hemida et al., 2021c, Hemida et al., 2020a, 2020b). However, neither the viral particles nor its nucleic acids were detected in the urine of infected camels (Farag et al., 2019). Meanwhile, recent studies reported the absence of any detectable viral nucleic acids of MERS‐CoV in some ticks infesting positive and actively shedding animals (Hemida et al., 2021b). Another recent study suggested that rodents sharing the habitats with an index MERS‐CoV naturally infected dromedary camel herd are unlikely to transmit the virus (Hemida et al., 2021a). Arthropods play important roles in both the mechanical and biological transmission of several viruses such as Yellow fever virus (YFV), Dengue haemorrhagic fever, Rift valley fever (RVFV), Chikungunya virus (CHIKV), West Nile virus (WNV) and Zika virus (Huang et al., 2019). The continuous transmission of some arthropod viruses in the environment is maintained by the interplay between the host, the vector and the environment (Diaz et al., 2012). Some viruses have one vector in their transmission cycle like YFV, CHIKV and RVFV while other viruses have several vectors in their transmission cycle such as WNV (Diaz et al., 2012). In the mechanical transmission, the insect carries the virus in their body parts then transfers to another host to touch the food, water or even touching the mucous membranes of this host. (Foil et al., 1983; Hoch et al., 1985) This will lead to the transmission of the virus from one host to another without any biological changes of the virus reported in the body of the insect as in the case of equine infectious anaemia virus (EIAV) and RVFV (Foil et al., 1983; Hoch et al., 1985). In the biological transmission of arthropod viruses, the arthropod sucks the blood of the virally infected host then transmitted it to a new host through biting. The virus replicates inside the midgut of the insect before it transmits to another host (Diaz et al., 2012; Kuno & Chang, 2005).

However, the roles of various species of arthropods in the transmission cycle of MERS‐CoV were never investigated yet.

## 2 MATERIALS AND METHODS

### 2.1 Description of the target dromedary camel population

**FIGURE 1:**
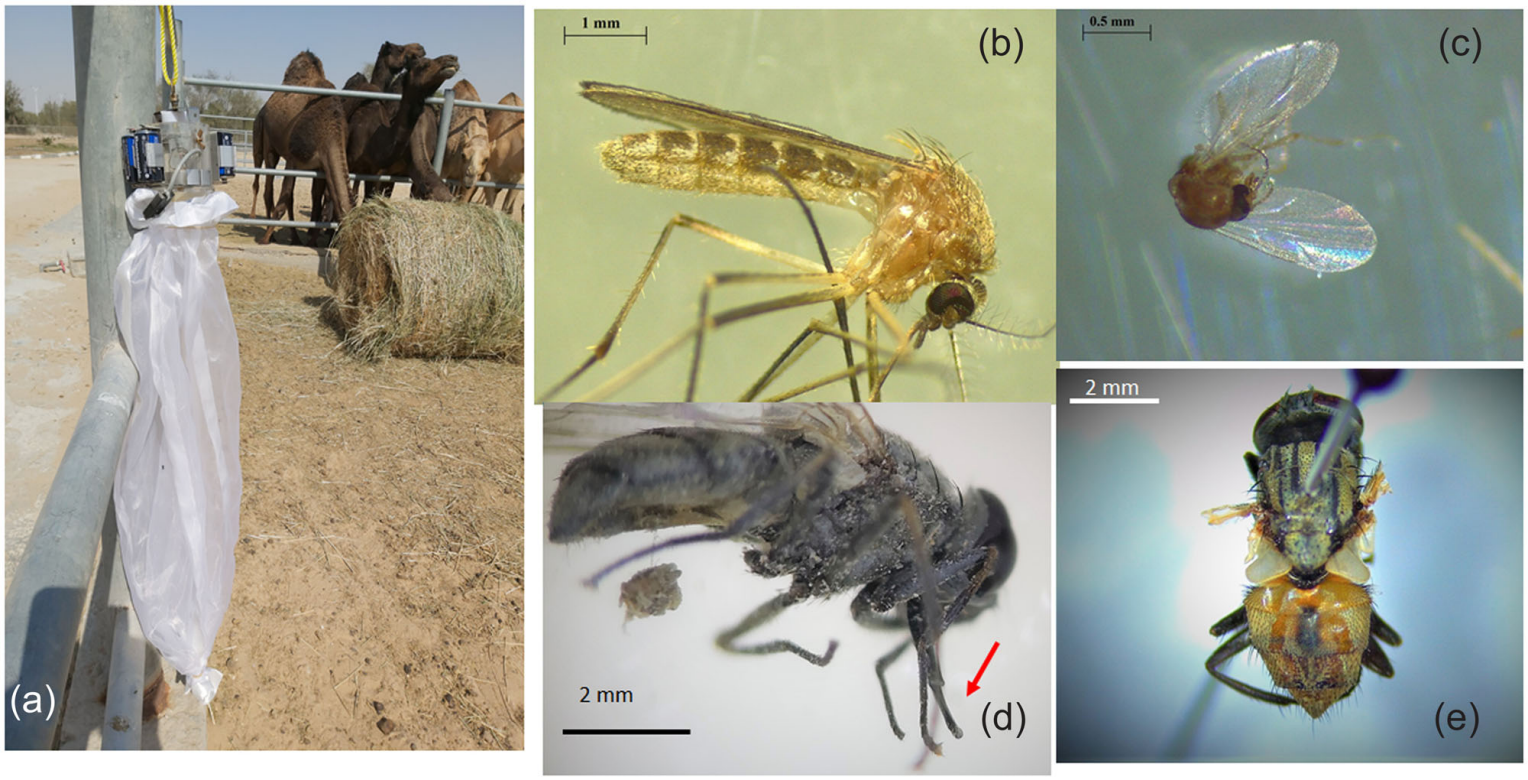
Morphology of some collected arthropods shared the habitat of MERS‐CoV naturally infected dromedary camel. (a) Typical CDC insect trap installed in the close proximity of the indexed MERS‐CoV positive dromedary camel population. (b) Photo for the *Culex theileri* species. (c) Photo for *Culicoides ibriensis* speceis. (d) Photo for the *Stomoxys calcitrans*. Note the piercing mouthpart (red arrow). (e) Photo for the *Musca domestica calleva*

Longitudinal molecular surveillance was conducted among a dromedary camel population for the MERS‐CoV (20 Feb to 20 April 2018). We identified an index MERS‐CoV naturally infected dromedary camel herd to conduct this study. These animals were kept in a 6000 square feet wire fence farm which consists of several animal pens separated by strong metal pipes (Figure 1). This farm was surrounded by some large cattle herds and some small ruminant herds separated by wire mesh. The adult male animals were kept in a separate pen and allow to approach the females during the breeding seasons. Animal sampling from the nasal swabs was carried out every two weeks until the end of this study.

### 2.2 Collection and preparation of tissue suspensions from the arthropods

We observed some species of arthropods such as the biting midges (*Culicoides*), the stable fly (*Stomoxys*), the house fly (*Musca domestica*) and some mosquitoes particularly (*Culex sp*) shared the habitat with the dromedary camel farm during the tenure of the current study (Figure 1). Table 1 is showing the number of the collected arthropods from each species at various intervals.

**TABLE 1:** Summary of the experimental design, sample collection, and results of the molecular surveillance among some arthropods sharing the habitat with MERS‐CoV naturally infected dromedary camels

Sp/ batches	I	II	III	IV	V	VI	VII
**Results of the MERS‐CoV molecular surveillance**							
**Dromedary camels**	15/40	9/15	9/9	9/9	9/9	7/9	6/9
**No. of tested species of arthropods per batch of collection**							
***Musca domestica***	20	30	25	30	20	20	15
***Stomoxys***	11	10	15	10	17	12	6
***Culex***	12	19	13	15	7	11	10
***Culicoides***	7	9	12	9	8	7	12

### 2.3 Insects collection methods

We used the CDC insect traps for the collection of the circulating strains of insects around the positive MERS‐CoV dromedary camel population. These traps were placed in very close vicinity of these camels, around their feeding and drinking trays. This trap contains a light source to attract the insects as previously described (Cohnstaedt et al., 2008). When a nocturnal insect flies near the bulb, it is drawn into a collection bag or killing jar by a downward current of air propelled by a fan mounted below the light source. We examined each trap early in the morning to makes sure the trapped insects were still alive to preserve the RNA integrity for the downstream testing.

We sorted the collected arthropods and then merged each species into separate 15 ml sterile conical tubes per batch of collection. We immediately placed the collected arthropods in an ice tank then transferred them to our laboratory. We added RNAlatter to the collected arthropods in each tube then placed them at (–80°C) for further processing. In the laboratory, we treated each individual whole insect as a separate sample in a separate tube to avoid any type of contamination. We washed the collected insects several times with sterile PBS. Briefly, each inset was placed in a 5 ml sterile tube containing 2 ml of PBS then homogenized tissue lyser by using some copper beads. The homogenized tissue suspensions were clarified by centrifugation at 500 RPM for 5 min at 4°C. The supernatants were collected and stored at (–80°C) for further testing. The extracted RNA concentration was carried out using the NanoDrop machine (Thermo Fisher Scientific/Applied Biosystems). The collected RNAs were stored at (–80°C) for further testing. The collection of mosquitoes was carried out into batches in parallel to animal sampling. Table 1 is showing the number of the sampled animals per batch as well as the number of each species of arthropods.

### 2.4 Collection and processing of dromedary camel's nasal swabs

We collected nasal swabs from the dromedary camel population for six‐time points biweekly during the duration of this study. Each tube of swab contains 3 ml of the viral transport medium [Kangjian Virus Collection and Preservation System (Cat: KJ502‐28‐1)]. The collected swabs were placed immediately in the ice‐containing tanks provided with a data logger to monitor the temperature. The collected swabs were usually transferred to our laboratory within 30 min of the collection time. Swabs were introduced deep into the nasal orifices of each animal to touch the inner mucous membranes. Special attention was paid that each cotton swab is fully soaked with the nasal secretions per each animal. Each swab was transferred into tubes containing the viral transport medium. The total viral RNAs were extracted from the nasal swabs from dromedary camels by using the Qiagen viral RNA kits (RNeasy Mini Kit, Qiagen, Hilden, Germany). The process of these extractions was carried out as previously described (Huang et al., 2018). Simply, 140 μl per sample was used to extract the total viral RNAs. The extracted RNAs were eluted in 50 μl of the elution buffer then were kept at (–80°C) for further testing.

### 2.5 Testing dromedary camel samples and suspensions from arthropods by the commercial MERS‐CoV‐real‐time‐PCR assay (qRT‐PCR)

We tested the nasal swabs collected from dromedary camels as well as the suspensions prepared from each species of arthropods by real‐time PCR. Briefly, we used the commercially available real‐time PCR MERS‐CoV kits (RealStar^®^ MERS‐CoV RT‐PCR Kit 1.0, Altona Diagnostics GmbH, Hamburg, Germany) to test various samples including nasal swabs and tissue suspensions prepared from various species of insects mentioned earlier for the presence of the viral nucleic acids. Several sets of control samples were used including some known negative and positive MERS‐CoV RNA samples. Samples were considered positive only when both targets (ORF1a and UpE) showing positive results. Meanwhile, we considered positive samples when (Ct ≤30).

## 3 RESULTS

### 3.1 Identification and classification of the circulating species of insects infesting and sharing the habitat with some MERS‐CoV‐positive camel population

The circulating species of insects in this farm reported in this study were shown to feed on animals’ blood and/or touching their mucous membranes especially mouse, nose, vagina and eyes. Classification of the insects revealed the presence of (*Culex theileri*, *Culicoides ibriensis, Stomoxys calcitrans* and *Musca domisctica calleva*.

### 3.2 Results of the molecular surveillance for the longitudinal study in dromedary camel population

Our MERS‐CoV molecular surveillance among this dromedary camel population showing that 40/52 animals were tested at the first batch of screening (Table 1). The results of the first batch showed 15/40 animals were positive. We selected nine animals out of those 15 showing the lowest Ct values for the follow‐up study as an indicator of the virus circulation in this herd. Results from the subsequent testing of the nine animals revealed that (9/9, 9/9, 9/9, 7/9 and 6/9) were positive at batches II–VII, respectively (Table 1). The Ct values per each animal from the selected nine animals and their age, sex and breed are listed in Table 2.

**TABLE 2:** Summary of animal information and the obtained q‐RT‐PCR‐Ct values for the follow‐up study on the selected nine animals

Animal	Age/y	Sex	Breed*	Batch/Ct values				
				III	IV	V	VI	VII
1	3	F	Magheem	19	23	22	26	27
2	2.5	F	Magheem	21	27	26	28	26
3	4	F	Magheem	18	25	28	27	28
4	3.5	F	Omani	24	25	27	29	29
5	2	F	Magheem	25	26	28	26	28
6	5	M	Sofor	29	28	25	37	38
7	2.5	F	Wodoah	16	19	24	27	28
8	8	F	Magheem	23	22	28	28	40
9	7	M	Magheem	28	29	27	38	39

* Breed classification based on the colour coat. F = female, M = male.

### 3.3 Results of the molecular surveillance of MERS‐CoV in some arthropods live in the proximity of some naturally infected dromedary camel population

Our molecular surveillance among various species of arthropods shares the habitat with the MERS‐CoV naturally infected dromedary camel showing the absence of any detectable MERS‐CoV‐RNAs in the extracted suspension of arthropods (Table 1).

## 4 DISCUSSION

Despite the great efforts in combating and mitigation of MERS‐CoV (Hemida & Alnaeem, 2019) since its emergence in 2012 (Zaki et al., 2012), there are ongoing reports for new cases from time to time (WHO, 2021; WHO, 22021). High seroprevalence and seroconversion of dromedary camels to MERS‐CoV were reported in camel populations in the Arabian Peninsula and Africa (Chu et al., 2018; Usman et al., 2020; Zhang et al., 2019). Several longitudinal studies conducted on some dromedary camel herds revealed the possibility of reinfection with MERS‐CoV (Hemida et al., 2017). This may be the reason behind the high level of antibodies in sera of some animals, especially in older animals above 2 years old (Nowotny & Kolodziejek, 2014). Some progress has been made to our understandings of the transmission of MERS‐CoV between animals and from animals to humans (Azhar et al., 2014; Farag et al., 2019; Hemida et al., 2021c, 2020b). However, there are many unknown possible avenues for virus transmission. Searching for some new unknown reservoir/s that may contribute to the sustainability and enhance the continuous transmission of the virus among dromedary camels is still one of the main research priorities in the epidemiology of MERS‐CoV. Some recent studies showed the lack of detection of MERS‐CoV‐RNAs in some species of ticks infesting some MERS‐CoV naturally infected camels (Hemida et al., 2021b). Another recent study reported the lack of detection of MERS‐CoV‐RNAs in samples from some species of rodents that shared their habitat with some index positive camel population (Hemida et al., 2021a). There are many faunas (mosquitoes, flies, ticks etc.) that live close to dromedary camels and sharing their habitats with them (Alahmed et al., 2019). Although various species of arthropods play important roles in the transmission cycle of many viruses (Failloux & Moutailler, 2015), their roles in the transmission of coronaviruses particularly MERS‐CoV are not well studied yet. The main objective of the current study was to investigate the potential roles of some species of arthropods in the transmission of MERS‐CoV. Our longitudinal study in the dromedary camel population revealed the circulation of the virus during the duration of this study. We sampled animals a 14 days’ time intervals. This was to make sure that MERS‐CoV is still circulating in the camel population during the tenure of the study. This is to ensure that the tested mosquitoes are in close contact with the positive MERS‐CoV camel population.

Arthropods may transmit viruses either mechanically or biologically or both (Fenner & Day, 1952). Some of these arthropods particularly mosquitoes*, culicoides* and *Stomoxys* suck the blood of animals and transfer it from one animal to another. Meanwhile, the housefly (*Musca domestica*) and *stomoxes* touch the mucus membranes of animals and may touch the saliva and other body secretions of dromedary camels. It was also noted that there is always a dramatic increase in the numbers of these arthropods during the summer particularly the *Musca domestica*. Each region may have its own species of arthropods that infest dromedary camels such as some species of ticks *Hyalomma impeltatum* (*H. impeltatum*) from Riyadh, Al‐Qaseem and Jazan regions (Al‐Khalifa et al., 2007). Sandfly plays important role in the transmission of the Rift valley fever virus (Hoch et al., 1984). Sandfly had been identified in the Arabian peninsula in Northern Saudi Arabia (Haouas et al., 2017).

All these factors may favour the transmission of some pathogens within a certain animal population including viruses. One of the interesting research directions is to collect some internal organs per each species of arthropods species particularly the salivary glands and guts and test them for the presence of MERS‐CoV or the viral nucleic acids. Taken together all this evidence into consideration, we conclude that arthropods at least the tested species in our study are unlikely to play roles in the transmission of MERS‐CoV. Some future large‐scale studies involving a large number of dromedary camel herds and other species of arthropods are required to explore/confirm the roles of various species of arthropods in the transmission of MERS‐CoV.

## 5 CONCLUSIONS

Our results are clearly showing the absence of any detectable MERS‐CoV‐RNA in tissue suspensions prepared from some species of arthropods living in close contact with some MERS‐CoV naturally infected dromedary camels. Thus, arthropods are unlikely to play roles in the transmission of MERS‐CoV.

## ANIMAL ETHICS STATEMENT

The animal experiments conducted in the current study were conducted as per the instructions of the Animal Ethics protocols and the National Committee of Bio‐Ethics, King Abdul‐Aziz City of Science and Technology, Royal Decree No. M/59 (http://www.kfsh.med.sa/KFSH_WebSite/usersuploadedfiles%5CNCBE%20Regulations%20ENGLISH.pdf). The animal experiments and protocols were reviewed and approved by the animal ethics committee of the deanship of scientific research, King Faisal University, Saudi Arabia (Approval No: KFU‐REC/2020‐09‐21). All the necessary paperwork for sample collections was obtained.

## CONFLICT OF INTEREST

The authors declare no conflict of interest.

### PEER REVIEW

The peer review history for this article is available at https://publons.com/publon/10.1002/vms3.717

## FUNDING

We wish to thank King Abdul‐Aziz City for Science and Technology (KACST), Saudi Arabia, for their generous funding through the MERS‐CoV research grant program (number 20‐0004/24‐1), which is a part of the Targeted Research Program (TRP). The funding body does not have any role in the design of experiments and the execution of the experiments.

## AUTHOR CONTRIBUTIONS

**Maged Hemida**: Conceptualization; Data curation; Formal analysis; Funding acquisition; Investigation; Methodology; Project administration; Resources; Software; Supervision; Validation; Visualization; Writing original draft; Writing review & editing. **Mohammad Nafi Solaiman Al‐Sabi**: Data curation; Formal analysis; Methodology. **Mohammed Alhammadi**: Conceptualization; Data curation; Methodology. **Faisal Almathen**: Conceptualization; Data curation; Formal analysis; Methodology. **Abdelmohsen Alnaeem**: Conceptualization; Formal analysis; Investigation; Methodology

## Data Availability

Data available on request from the authors.
